# Effects of Iodine Doping on Electrical Characteristics of Solution-Processed Copper Oxide Thin-Film Transistors

**DOI:** 10.3390/ma14206118

**Published:** 2021-10-15

**Authors:** Hyeonju Lee, Xue Zhang, Bokyung Kim, Jin-Hyuk Bae, Jaehoon Park

**Affiliations:** 1 Department of Electronic Engineering, Hallym University, Chuncheon 24252, Korea; hyeonjulee@hallym.ac.kr (H.L.); D21012@hallym.ac.kr (B.K.); 2 College of Ocean Science and Engineering, Shangdong University of Science and Technology, Qingdao 266590, China; skd996027@sdust.edu.cn; 3 School of Electronics Engineering, Kyungpook National University, Daegu 41566, Korea

**Keywords:** iodine doping, copper oxide semiconductor, solution process, thin-film transistor

## Abstract

In order to implement oxide semiconductor-based complementary circuits, the improvement of the electrical properties of p-type oxide semiconductors and the performance of p-type oxide TFTs is certainly required. In this study, we report the effects of iodine doping on the structural and electrical characteristics of copper oxide (CuO) semiconductor films and the TFT performance. The CuO semiconductor films were fabricated using copper(II) acetate hydrate as a precursor to solution processing, and iodine doping was performed using vapor sublimated from solid iodine. Doped iodine penetrated the CuO film through grain boundaries, thereby inducing tensile stress in the film and increasing the film’s thickness. Iodine doping contributed to the improvement of the electrical properties of the solution-processed CuO semiconductor including increases in Hall mobility and hole-carrier concentration and a decrease in electrical resistivity. The CuO TFTs exhibited a conduction channel formation by holes, that is, p-type operation characteristics, and the TFT performance improved after iodine doping. Iodine doping was also found to be effective in reducing the counterclockwise hysteresis in the transfer characteristics of CuO TFTs. These results are explained by physicochemical reactions in which iodine replaces oxygen vacancies and oxygen atoms through the formation of iodide anions in CuO.

## 1. Introduction

Oxide semiconductors have been used as active channel materials in thin-film transistors (TFTs) owing to their excellent charge carrier mobility, high optical transmittance in the visible range, excellent chemical stability, and versatility in processing. Therefore, oxide semiconductor-based TFTs have been used in various electronic applications such as electronic memory devices, chemical sensors, and active matrix displays [1,2,3].

To date, oxide TFTs mostly exhibit n-channel operation behavior because n-type semiconductors, such as zinc oxide, indium oxide, indium zinc oxide, and indium gallium zinc oxide, are widely used [4,5,6,7]. However, p-channel oxide TFTs have rarely been reported because of complicated fabrication procedures. The local distribution of anisotropic oxygen 2p orbitals is a main factor in determining the valence band maximum of p-type oxide semiconductors, which results in large effective mass and low mobility for holes in p-type oxide semiconductors [8,9,10]. For this reason, a transparent p-type copper iodide (CuI) semiconductor has recently been proposed as a replacement for p-type oxide semiconductors [11]. However, its excessive hole concentration (>10^19^ cm^−3^), due to metal vacancies, lowers the on/off current ratio of the TFT to less than 10^2^, which inevitably causes a significant problem in degrading the switching function of the transistors; the pristine CuI semiconductor should thus be doped with metal ions, such as Zn^2+^, Ga^3+^, and Sn^4+^ [12]. Although it is difficult to realize p-type high-performance oxide TFTs, oxide semiconductor-based complementary circuits and p–n junction systems still need to be demonstrated [8,13]. Here, we think it is important to point out that p-type oxide semiconductors are more likely to form binary, ternary, and quaternary compositions, compared to CuI. The diversity in the chemical composition of p-type oxide semiconductors is essentially advantageous in applications to electronic devices because the electrical properties of the p-type oxide semiconductor can be adjusted according to the chemical composition. Among p-type oxide semiconductors, Cu-based oxides are relatively simple to fabricate, and they also exhibit promising electro-optical properties. These features establish suitability for p-type semiconductor applications. Recent studies have mostly focused on copper oxide (CuO) semiconductors for electronics and energy devices because it offers the advantages of non-toxicity, cost-effectiveness, and abundance. To meet the demand for high-performance p-channel devices, Bae et al. enhanced the performance of copper-based TFTs by doping the semiconductor film with gallium atoms to reduce oxygen vacancies, which are known to interfere with the conduction of hole carriers [14]. Moreover, Baig et al. demonstrated that the doping of CuO with yttrium atoms could enhance the device performance [15]. The doping process for p-type oxide semiconductors, as demonstrated in previous studies, is important to modulate the charge carrier density of the semiconductor film and improve the electrical performance of CuO-based TFTs. Nevertheless, research on doping technology for p-type oxide semiconductors is still in its infancy and doping these materials as a post-processing technology has hardly been studied.

In the present paper, we report the effects of iodine doping on the structural and electrical characteristics of solution-processed CuO semiconductor films and the TFT performance. Note that solution processing is a simple and cost-effective method for fabricating oxide semiconductors. Here, the p-type CuO semiconductor films were formed via spin-coating and the CuO film was doped with iodine vapor. Experimental results demonstrated that iodine doping can be a novel post-processing method to improve the electrical properties of CuO semiconductors and the performance of CuO TFTs.

## 2. Materials and Methods

**Figure 1:**
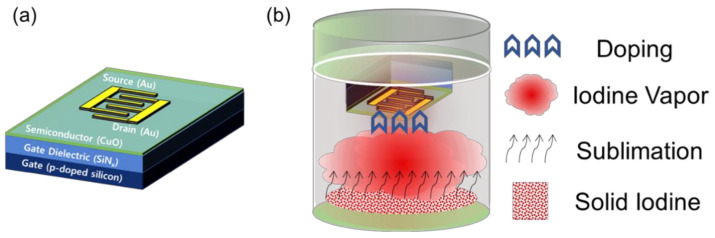
Schematic representations of (**a**) the fabricated CuO TFT and (**b**) the iodine-doping installation.

To fabricate CuO TFTs with an inverted staggered structure shown in Figure 1a, a p-doped silicon substrate with a 100-nm-thick silicon nitride (SiN_x_) dielectric layer was sequentially cleaned by sonication in acetone, isopropyl alcohol, and deionized water. Oxygen plasma treatment was performed to make the substrate surface hydrophilic, which was a necessary process to improve the coatability of the CuO precursor solution on the substrate. For the oxygen plasma treatment, a radio frequency power of 45 W was applied for 2 min, while the oxygen flow rate was maintained at 20 sccm. The CuO-precursor solution was prepared by dissolving 0.3 M of copper(II) acetate hydrate [Cu(CO_2_CH_3_)_2_H_2_O] in 2-methoxyethanol, which was then stirred using a magnetic bar at a rotation speed of approximately 750 rpm on a hotplate (Conring, Seoul, Korea) heated to 75 °C for 1 h. The precursor solution was filtered through a poly-tetrafluoroethylene syringe filter (Hyundai Micro, Seoul, Korea) with pore size of 0.2 μm and spin-coated on the oxygen-plasma-treated substrate at 2000 rpm for 1 min. The coated film was dried on a hotplate at 80 °C for 5 min and 120 °C for 20 min to evaporate the solvent and then thermally annealed in a vacuum tube furnace (Daeki, Daejeon, Korea) at 500 °C for 30 min. Finally, 30-nm-thick Au source and drain electrodes with an interdigitated geometry were thermally deposited on the CuO semiconductor layer through a shadow mask under a base pressure of approximately 6 × 10^−6^ Torr. The interdigitated electrodes consisted of 5 pairs with a 80-µm channel length and a 400-µm electrode width. Thus, the effective channel length and width in our transistors were 80 µm and 2000 µm, respectively. To dope the CuO films with iodine, the fabricated CuO TFTs were exposed to iodine vapor for 5 s as shown in Figure 1b; iodine vapor was produced by sublimation from solid iodine at room temperature, and the vapor pressure of iodine at room temperature is known to be approximately 0.4 mbar.

In this study, a thermogravimetric analysis (TGA) was carried out to examine the thermal decomposition behavior of the precursor solution. The influence of iodine doping on the morphological and structural characteristics of the fabricated CuO films was investigated using atomic force microscopy (AFM) (XE150, PSIA, Santa Clara, CA, USA) and field emission scanning electron microscopy (FE-SEM) (JEOL, Tokyo, Japan). Contact angle measurement using deionized water drop was performed to evaluate the change in the surface wettability of the CuO films. Raman spectroscopy was used to investigate the influence of iodine doping on the lattice structure of CuO films. The Hall effect measurement using van der Pauw method was conducted to measure the Hall mobility, electrical resistivity, and carrier concentration of the CuO films. Electrical characteristics of the fabricated TFTs were measured in a dark box under ambient air conditions.

## 3. Results and Discussion

To analyze the thermal decomposition process of the prepared CuO precursor solution, a thermogravimetric measurement of the solution was performed in nitrogen ambient by increasing the temperature from 25 °C to 700 °C at a heating rate of 10 °C·min^−1^.

**Figure 2:**
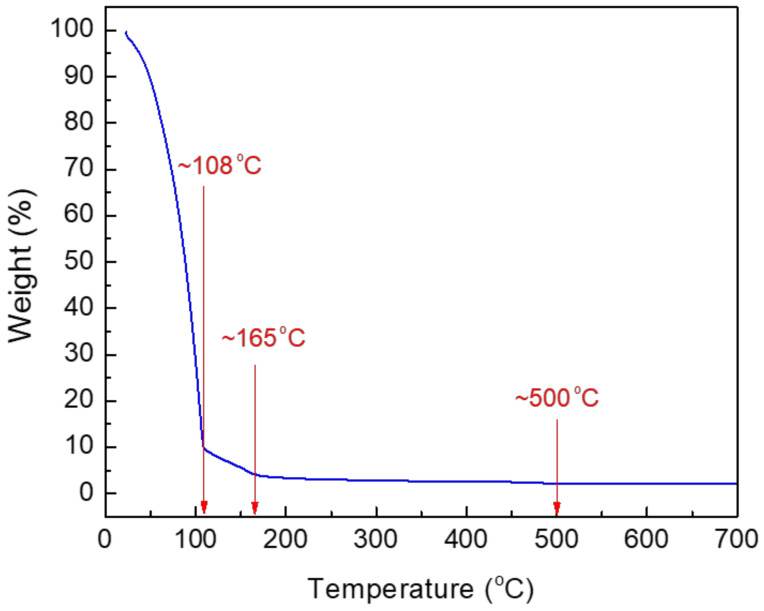
TGA characteristic curve of the prepared CuO precursor solution.

Figure 2 shows the measured TGA curve of the CuO precursor solution. As the temperature increased to approximately 108 °C, most of the weight loss (>90%) of the precursor solution occurred because of the evaporation of the 2-methoxyethanol solvent. As the temperature increased from 108 °C to 165 °C, the rate of weight loss gradually decreased. This implies that Cu(CO_2_CH_3_)_2_H_2_O is hydrolyzed to Cu(OH)_2_. As the temperature exceeded 165 °C, the rate of weight loss reduced further, and the dehydration reaction of Cu(OH)_2_ to form CuO is thought to have occurred in this temperature range. In particular, there was no discernible change in weight at temperatures above 500 °C. Accordingly, the optimal annealing temperature to fabricate the CuO semiconductor films in this experiment was determined to be 500 °C.

**Figure 3:**
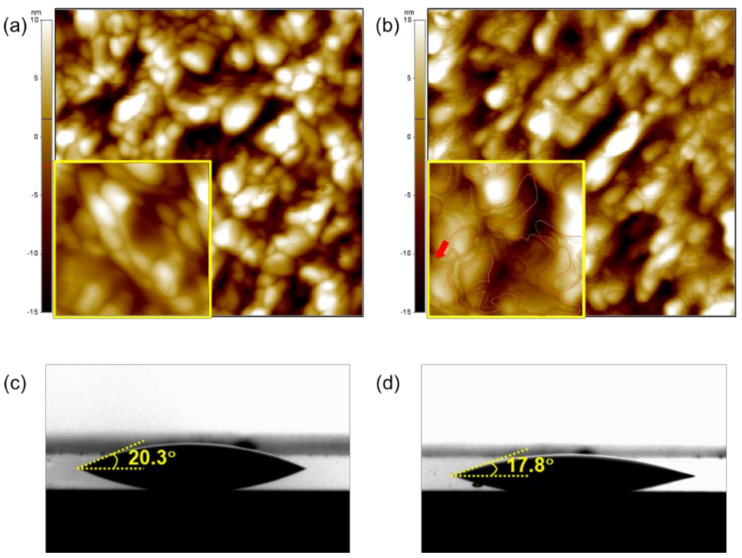
AFM images (1 μm × 1 μm) of (**a**) pristine and (**b**) iodine-doped CuO films. Insets show the enlarged AFM images (0.3 μm × 0.3 μm). Photographs of water droplets on the surfaces of (**c**) pristine and (**d**) iodine-doped CuO films.

Figure 3a,b show the surface morphologies of the pristine and iodine-doped CuO films, which were characterized using AFM. Both of the films had similar surface morphologies, suggesting that iodine doping did not cause significant morphological changes, such as pore formation, delamination, or cracking in the solution-processed CuO film. However, the iodine-doped CuO film had a rather smooth surface compared to the pristine CuO film; the root-mean-square roughness values of the pristine and iodine-doped CuO films were approximately 6.7 ± 0.3 nm and 6.1 ± 0.2 nm, respectively. The area marked with a red line in the inset of Figure 3b represents the area where iodine was supposed to exist, especially at the grain boundaries, which suggests that iodine mostly penetrates the CuO film through grain boundaries. Therefore, the decrease in the surface roughness of the iodine-doped CuO film may be due to the iodine remaining at the grain boundaries while iodine penetrates into the CuO film through grain boundaries. Figure 3c,d show that water is wetting the pristine CuO film with a contact angle of approximately 20.3°, while the contact angle of water drop on the iodine-doped CuO film is approximately 17.8°. The change in contact angles of water drop is also thought to be a result of the iodine remaining at grain boundaries during the doping process of the CuO film, as observed in the AFM images.

**Figure 4:**
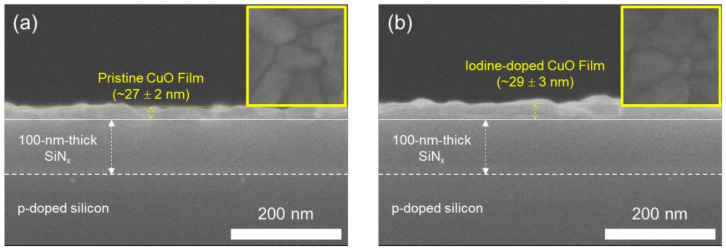
Cross-sectional FE-SEM images of (**a**) pristine and (**b**) iodine-doped CuO films. Insets show the top-view FE-SEM images (100 nm × 100 nm) of films.

Figure 4 shows the cross-sectional FE-SEM images of pristine and iodine-doped CuO films; the CuO films were formed on a p-doped silicon substrate having a 100-nm-thick SiN_x_ dielectric layer. In our results, the iodine-doped CuO film (thickness ~29 ± 3 nm) was slightly thicker than the pristine CuO film (thickness ~27 ± 2 nm), indicating the penetration of iodine into the CuO film. The insets show the FE-SEM surface images of the CuO films. As shown in the insets of Figure 4, the pristine and iodine-doped CuO films exhibited similar surfaces; CuO grains with a size of several tens of nanometers are packed in both the films. Based on the AFM and FE-SEM results, it is reasonable to state that iodine, which penetrates into the film through grain boundaries, increases the thickness of the CuO film.

**Figure 5:**
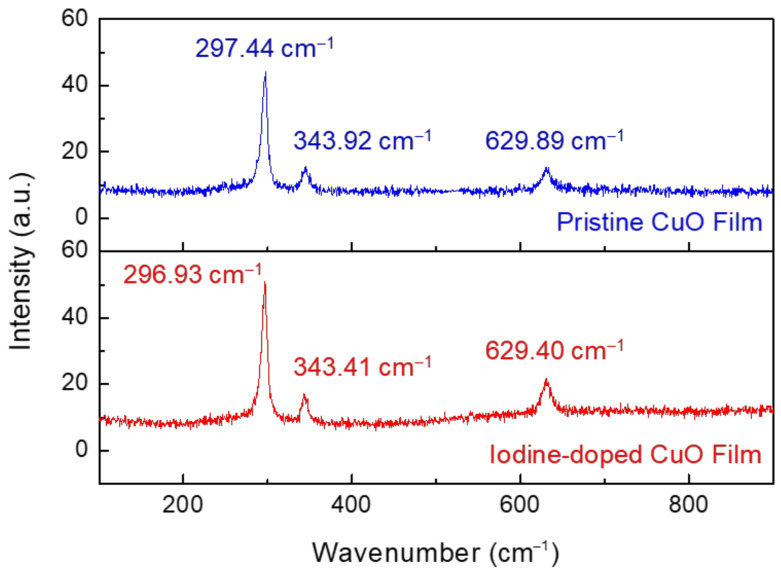
Raman spectra of pristine and iodine-doped CuO films.

We further investigated the influence of iodine doping on the lattice structure of CuO films using Raman spectroscopy; for the measurement, the wavelength of excitation laser beam was fixed at 532 nm and the laser spot size was controlled at approximately 1 μm. Figure 5 shows the Raman spectra of the solution-processed CuO films before and after iodine doping. The pristine CuO film exhibited Raman peaks at approximately 297.44 cm^−1^, 343.92 cm^−1^, and 629.89 cm^−1^, whereas the corresponding Raman peaks in the iodine-doped CuO film appeared at approximately 296.93 cm^−1^, 343.41 cm^−1^, and 629.40 cm^−1^. As these wavenumbers of Raman characteristic peaks are similar to those reported in the literature, we can assign the peak at 297.44 cm^−1^/296.93 cm^−1^ to the A_g_ mode and the peaks at 343.92 cm^−1^/343.41 cm^−1^ and 629.89 cm^−1^/629.40 cm^−1^ to the B_g_ modes of CuO [16,17]. Importantly, the iodine doping of CuO causes shifts in Raman peak positions towards the low wavenumbers. Considering that the tensile and compressive stresses can be characterized by shifts toward lower and higher wavenumbers [18,19], respectively, the shifts in the peak positions towards low wavenumber reveal that the CuO film underwent tensile stress due to the permeation of iodine into the film. The results of Raman spectroscopy indicate that iodine penetrating into the CuO film induces tensile stress in the film, thereby causing a change in lattice properties.

**Figure 6:**
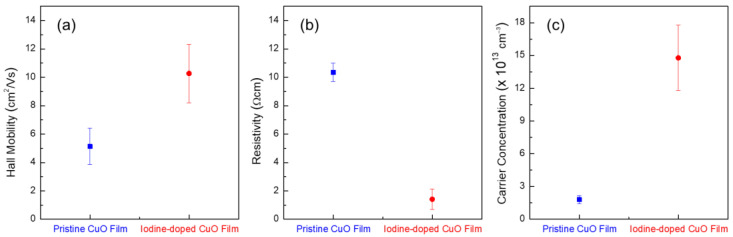
Hall effect measurements of pristine and iodine-doped CuO films. (**a**) Hall mobilities, (**b**) resistivities, and (**c**) hole-carrier concentrations.

The change in the lattice structure of the CuO film due to iodine doping may change the electrical properties of the film. Figure 6 compares the Hall mobility, resistivity, and hole-carrier concentration the CuO film before and after iodine doping. Iodine doping of the CuO film is observed to increase Hall mobility from 5.13 cm^2^·V^−1^·s^−1^ to 10.27 cm^2^·V^−1^·s^−1^ and decrease electrical resistivity from 10.35 Ω·cm to 1.41 Ω·cm. It is also found that the hole-carrier concentration in the CuO film could be increased from 1.78 × 10^13^ cm^−1^ to 14.73 × 10^13^ cm^−1^ due to iodine doping. These variations in Hall mobility, resistivity, and carrier concentration confirm that the structural modification of the solution-processed CuO films due to iodine doping can also enhance the electrical properties of the films.

**Figure 7:**
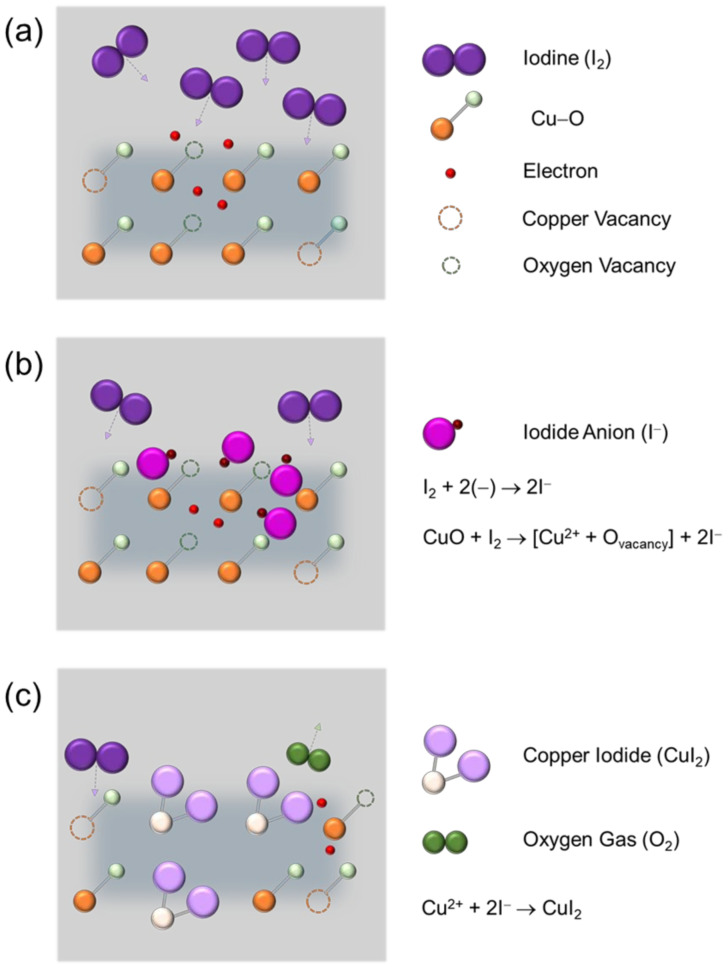
Possible reactions caused by iodine in the CuO film. (**a**) CuO film before iodine penetration, (**b**) formation of iodide anions after iodine penetration, and (**c**) formation of copper iodides.

In order to explain the change in the electrical properties of CuO semiconductor materials due to iodine doping, the reactions caused by iodine penetrating into the CuO film are assumed, as shown in Figure 7. Figure 7a shows the lattice structure of the pristine CuO film in which Cu–O bonds, copper vacancies, and oxygen vacancies exist. It is well known that copper vacancies generate holes that are majority charge carriers of the p-type CuO semiconductor, and oxygen vacancies generate electrons that are minority charge carriers. Moreover, Cu 3d orbitals and O 2p orbitals are hybridized to form a valence band of CuO, in which holes can move freely [20]. Figure 7b shows the case where iodine penetrates into the CuO film through iodine doping. Firstly, iodine penetrating the CuO film is expected to combine with electrons distributed in the film to form two iodide anions (I^−^). Secondly, another oxygen vacancies, i.e., extra electrons, may be generated in the film through the reduction reaction of CuO by iodine, and these extra electrons and iodine may combine to form additional iodide anions. Among these two processes that produce I^−^ anions, the latter process, which requires reduction in CuO, is likely to take longer time for I^−^ anion formation, as compared to the former process. As shown in Figure 7c, I^−^ anions generated by the above reactions combine with Cu^2+^ cations to form copper iodide (CuI_2_) and can also release oxygen (O_2_) gas. As a result, there is a possibility that I 5p orbitals are locally hybridized in the valence band of CuO, which is formed due to Cu 3d orbitals and O 2p orbitals, by replacing I_2_ with oxygen atoms or oxygen vacancies through iodine doping in the CuO film. Therefore, the presence of I 5p orbitals with a relatively larger size than O 2p orbitals in the CuO lattice can contribute to the delocalization of energy states for hole transport in the valence band of CuO, thereby increasing Hall mobility and improving electrical conductivity, that is, reducing electrical resistivity, as observed in the Hall effect results. It should be noted that the iodine atom (atomic diameter: approximately 115 pm) is larger than the oxygen atom (atomic diameter: approximately 48 pm). This may explain why the replacement of oxygen atoms or oxygen vacancies by iodine atoms causes an increase in the thickness of the CuO film, as confirmed in the FE-SEM cross-sectional images, and induces tensile stress in the CuO film, as observed in the Raman results. The proposed model also points to a decrease in electron and oxygen vacancy due to iodine doping of CuO. Accordingly, the change in the structural and electrical properties of the CuO film due to iodine doping can be understood through the physicochemical reactions caused by iodine.

**Figure 8:**
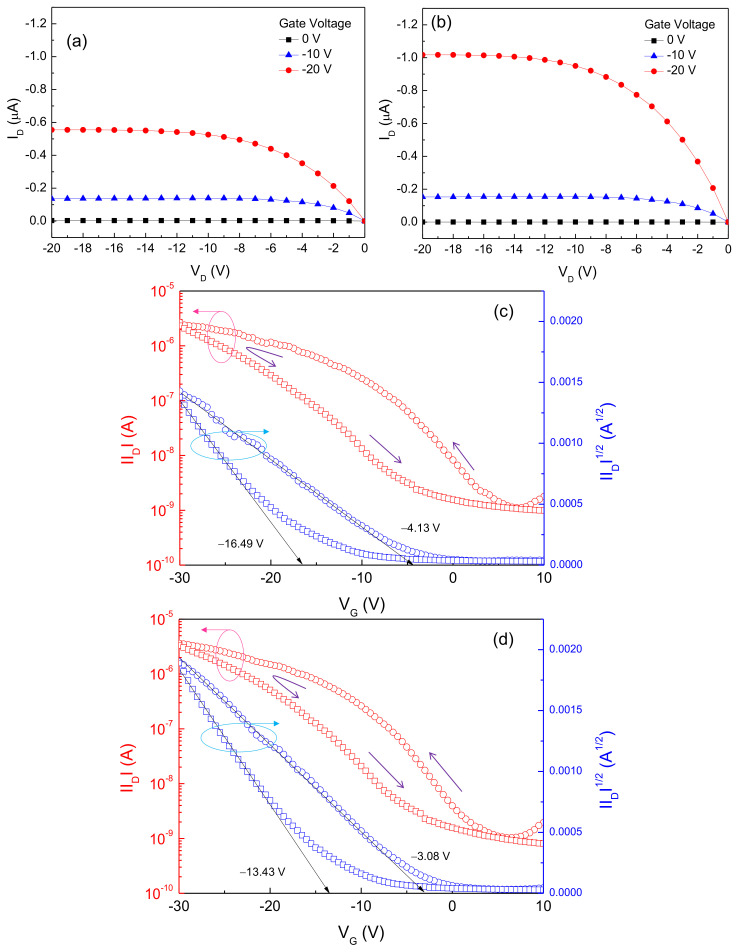
Output characteristics (I_D_ versus V_D_ plots) of the CuO TFT (**a**) before and (**b**) after iodine doping. Transfer characteristics of the CuO TFT (|I_D_| and |I_D_|^1/2^ versus V_G_ plots) (**c**) before and (**d**) after iodine doping.

To examine the effect of iodine doping on the performance of solution-processed CuO TFTs, the electrical characteristics of the transistor were measured before and after doping with iodine. Figure 8a shows the output characteristics of the CuO TFT, which were measured before iodine doping by changing the drain voltage (V_D_) from 0 V to −20 V in increments of −1 V at gate voltages (V_G_) of 0 V, −10 V, and −20 V. The CuO TFT exhibited a clear pinch-off and excellent saturation under p-channel accumulation mode operation, indicating that holes are majority charge carriers in the CuO semiconductor film. Moreover, an important observation from Figure 8b is that the output characteristics of the CuO TFT could be enhanced through iodine doping process. After iodine doping, the TFT exhibited higher drain currents (I_D_), while the pinch-off and saturation behaviors could still be maintained without degradation. Figure 8c,d show the transfer characteristics of the CuO TFT before and after iodine doping, respectively. These characteristics were measured at a fixed drain voltage of −20 V, while the gate voltage was swept reversibly from 10 V to −30 V in increments of −1 V. In order to evaluate the performance of TFT, the subthreshold swing (S.S.), which is defined as the change in the gate voltage required to change the drain current by a factor of 10, was extracted from the plot of |I_D_|versus V_G_, and the threshold voltage (V_T_) was obtained from the plot of |I_D_|^1/2^ versus V_G_ by extrapolating to the drain current of 0 A. The field-effect mobility (μ_eff_) was calculated in the saturation region. The TFT parameters are summarized in Table 1. When the gate voltage was swept from 10 V to −30 V, the CuO TFT without iodine doping exhibited a subthreshold swing of 3.3 V·decade^−1^, threshold voltage of −4.13 V, field-effect mobility of 4.25 × 10^−^^3^ cm^2^·V^−1^·s^−1^, and on/off current ratio of 2.4 × 10^3^. Upon reversing the gate voltage sweep direction from −30 to 10 V, the threshold voltage shifted in the negative direction and a counterclockwise hysteresis was observed; the measured shift in the threshold voltage was approximately −12.36 V. After iodine doping, the transistor exhibited a subthreshold swing of 3.0 V·decade^−1^, threshold voltage of −3.08 V, field-effect mobility of 6.61 × 10^−3^ cm^2^·V^−1^·s^−1^, and on/off current ratio of 3.51 × 10^3^. Considering that the field-effect mobility reported in the performance improvement study for the precursor-based solution-processed p-type CuO TFTs was approximately 2.83 × 10^3^ cm^2^·V^−1^·s^−1^ [21], the field-effect mobility of the iodine-doped CuO TFTs is quite remarkable. In addition, a negative shift in the threshold voltage and counterclockwise hysteresis were also observed upon reversal of the gate voltage sweep direction; the shift in the threshold voltage was approximately −10.35 V. The decrease in the threshold voltage and increase in the field-effect mobility by iodine doping could be due to the improvement of the electrical properties of the CuO semiconductor film, as confirmed by the Hall effect results in Figure 6. Herein, we should note that the counterclockwise hysteresis, the negative shift of the threshold voltage, is reduced by iodine doping. It is well known that counterclockwise hysteresis for p-type TFTs is caused by the mobile charges and defect-related trap states [22]. Based on our model in Figure 7, the reduction in electrons and oxygen vacancies by iodine doping is thus believed to contribute to reducing the hysteresis in the transfer characteristics of CuO TFTs.

**Figure 9:**
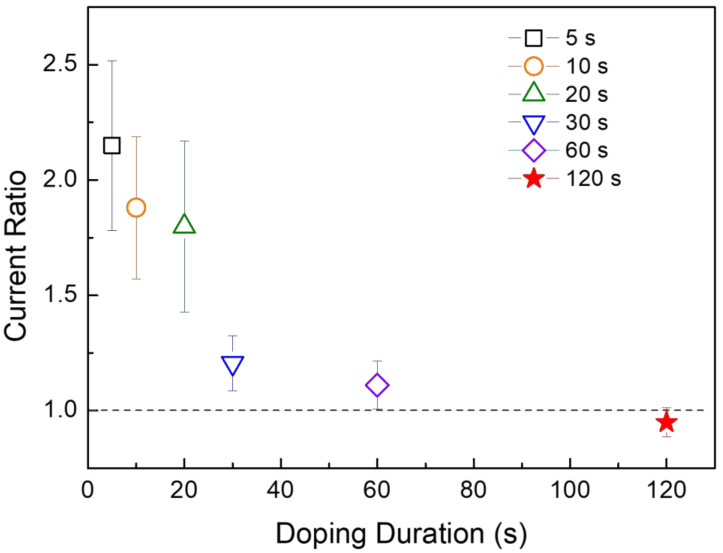
Comparison of current ratio properties according to iodine doping duration of the solution-processed CuO TFTs.

Additionally, we observed changes in the drain current of CuO TFTs while increasing the duration of iodine doping. Figure 9 shows a comparison of the changes in the drain current while varying the duration of iodine doping; the change in current was expressed as a current ratio obtained by dividing the drain current measured after iodine doping by the drain current measured before iodine doping, and at least ten TFTs were used under each condition to examine the effect of iodine-doping duration. Importantly, the current ratio is found to decrease with an increase in iodine-doping duration. When the duration of iodine doping was 120 s, the drain current of the TFT deteriorated after doping, as indicated by a current ratio of less than 1.0. This means that there is a limit to improving TFT performance even if the iodine-doping duration is increased. We consider that the physicochemical reactions of iodine during long-duration doping process may deteriorate the energy states for hole transport in the valence band of CuO by further augmenting the lattice deformation and tensile stress in the CuO semiconductor layer. According to the model we proposed (Figure 7), iodine doping for a long duration can significantly increase the reduction reaction of CuO, which greatly reduces Cu–O bonds in the lattice structure. Quantitative analyses of the energy characteristics of CuO, such as density of states and carrier distribution in the energy band structure, as a function of iodine concentration in the film is expected to contribute to optimization of the iodine doping process. Consequently, this study demonstrates that iodine doping represents a novel method to improve the electrical properties of CuO semiconductors in general and the performance of CuO TFTs in particular. For a comprehensive understanding of the physicochemical reaction mechanism by doped iodine in CuO, further studies are required to analyze the stoichiometric properties of iodine-doped CuO semiconductor films according to the spatial distribution of iodine.

## 4. Conclusions

We investigated the effects of iodine doping on the structural and electrical characteristics of p-type CuO semiconductors and the performance of CuO TFTs. The doped iodine penetrated the film, inducing tensile stress and increasing the thickness of the film. In addition, iodine doping contributed to increasing Hall mobility and hole-carrier concentration and decreasing electrical resistivity. According to the physicochemical reaction model by iodine proposed in this study, the replacement of oxygen atoms or oxygen vacancies by doped iodine induces delocalization of energy states for the transport of holes, which are majority carriers, in the valence band of CuO. This explains the improvement in the electrical properties of p-type CuO semiconductors through iodine doping, which, in turn, enhanced the TFT performance. In particular, it was found that the reduction in minority carrier electrons and oxygen vacancies in the CuO semiconductor film due to iodine doping is effective at reducing the hysteresis in the transfer characteristics of the transistor. Experimental results demonstrate that iodine doping, as a post-processing method, can be used to improve the electrical characteristics of p-type oxide semiconductor materials, thereby developing p-type oxide TFTs with high performance. Effects of iodine doping on the crystallinity of CuO semiconductors and the electrical stability of CuO TFTs remain to be studied. We believe that the physicochemical reactions by iodine proposed in this study provide a basis for further research pertaining to the development of various sensors fabricated using solution-processed oxide TFT-based circuits.

## Figures and Tables

**Table 1:** Summary of performance parameters for the pristine and iodine-doped CuO TFTs obtained by reversing the direction of V_G_ sweep.

-	S.S. [V·Decade^−1^]	V_T_ [V]	μ_eff_ [cm^2^·V^−1^·s^−1^]	On/Off Current Ratio
Pristine CuO TFT (V_G_: 10 V → −30 V)	3.3	−4.13	4.25 × 10^−^^3^	2.40 × 10^3^
Pristine CuO TFT (V_G_: −30 V → 10 V)	5.1	−16.49	1.14 × 10^−^^2^	2.33 × 10^3^
Iodine-doped CuO TFT (V_G_: 10 V → −30 V)	3.0	−3.08	6.61 × 10^−^^3^	3.51 × 10^3^
Iodine-doped CuO TFT (V_G_: −30 V → 10 V)	4.7	−13.43	1.27 × 10^−^^2^	4.12 × 10^3^
